# Supplementary material for: Viscoelastic Properties of Water-Absorbed Poly(methyl methacrylate) Doped with Lithium Salts with Various Anions
Source: Molecules. 2022 Oct 21;27(20):7114. doi: 10.3390/molecules27207114 (PMC9607444; doi:10.3390/molecules27207114)
Supplement: Supplementary file 1 [file molecules-27-07114-s001.zip › molecules-1874905-supplementary.pdf]

**Supporting Information**

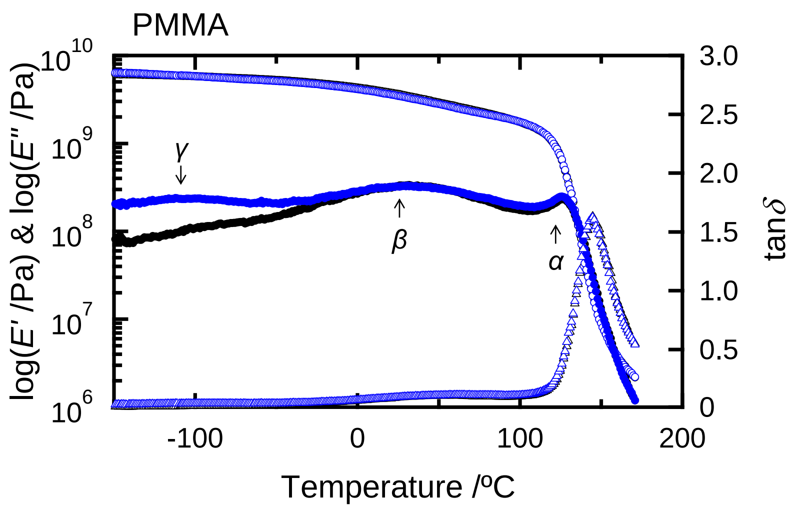


Figure S1. Temperature dependence of dynamic mechanical properties of PMMA.

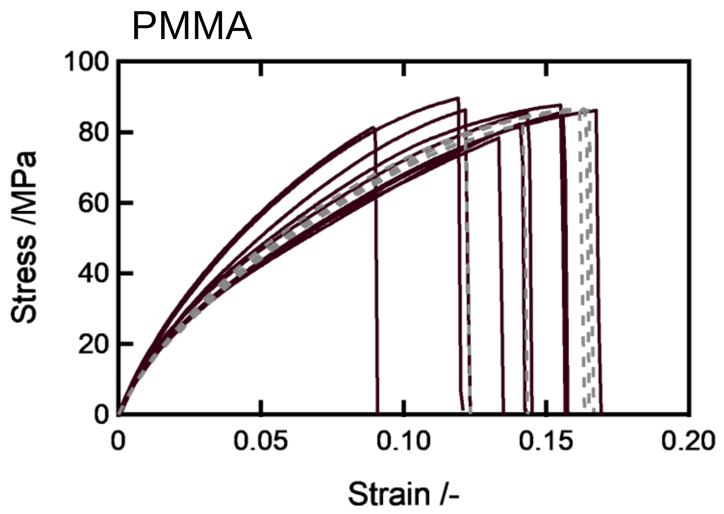


Figure S2. Stress-strain curves of dried and moisture pristine PMMA: dried (solid line) and moisture-absorbed (dashed line) samples.

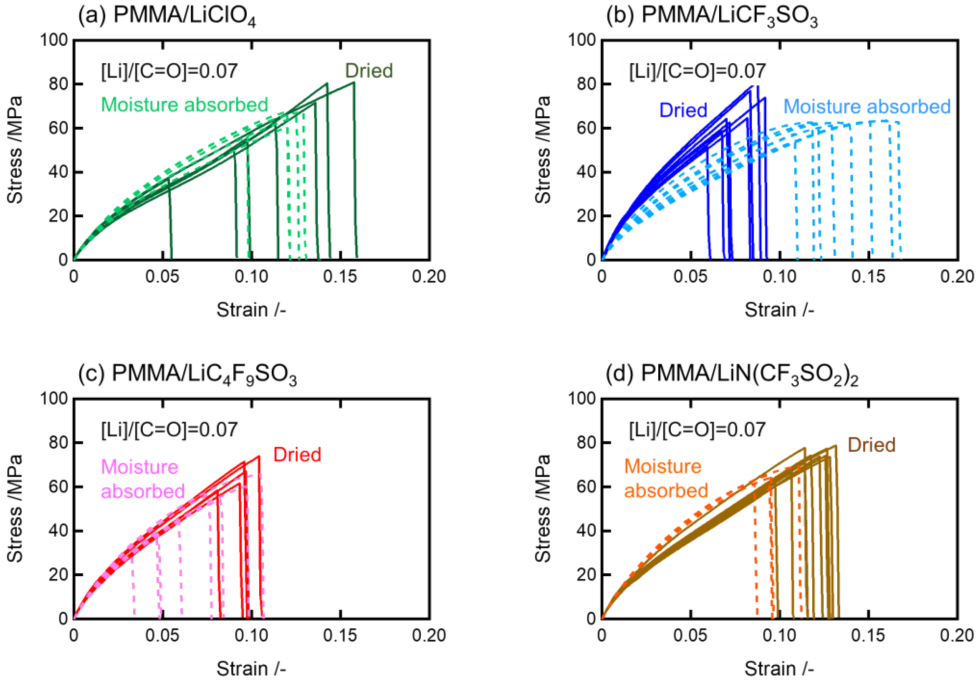


Figure S3. Stress-strain curves of dried and moisture PMMA/salt sheets with a 0.07 molar ratio salt concentration: dried (solid line) and moisture-absorbed (dashed line) samples.
